# Fundus Autofluorescence in Diabetic Retinopathy

**DOI:** 10.3390/jpm14080793

**Published:** 2024-07-26

**Authors:** Otilia-Maria Dumitrescu, Mihail Zemba, Daniel Constantin Brănișteanu, Ruxandra Angela Pîrvulescu, Madalina Radu, Horia Tudor Stanca

**Affiliations:** 1 Department of Ophthalmology, “Dr. Carol Davila” Central Military Emergency University Hospital, 010825 Bucharest, Romania; 2 Department of Ophthalmology, University of Medicine and Pharmacy “Carol Davila”, 050474 Bucharest, Romania; 3 Department of Ophthalmology, University of Medicine and Pharmacy “Grigore T. Popa”, 700115 Iasi, Romania; daniel.branisteanu@umfiasi.ro

**Keywords:** fundus autofluorescence, diabetic retinopathy, diabetic macular oedema

## Abstract

Diabetic retinopathy is a leading cause of visual morbidity worldwide. Fundus autofluorescence is a rapid, non-invasive imaging modality that has gained increased popularity in recent years in the multimodal evaluation of diabetic retinopathy and, in particular, of diabetic macular oedema. Acquired using either a fundus camera or the confocal scanning laser ophthalmoscope, short-wavelength and near-infrared autofluorescence are the most used techniques in diabetic retinopathy. In diabetic macular oedema, short-wavelength autofluorescence, in its cystoid pattern, is useful for detecting cystoid macular oedema. Increased spot hyperautofluorescence in short-wavelength and granular changes in near-infrared autofluorescence correlate well with other imaging findings, indicating photoreceptor and retinal pigment epithelium damage and being associated with decreased visual acuity. While also being a marker of oxidative stress, increased short-wavelength autofluorescence in the setting of diabetic macular oedema appears to be a prognostic factor for poor visual outcome, even after the resolution of the intraretinal fluid. Autofluorescence also helps in the assessment of diabetic retinal pigment epitheliopathy and choroidopathy. Fundus autofluorescence is an evolving technology that will assist in gaining further insight into the pathophysiology of diabetic retinopathy and allow for a more comprehensive evaluation of these patients.

## 1. Introduction

Diabetes mellitus (DM) affects an estimated 537 million adults worldwide, with type 2 diabetes accounting for 90% of all cases [1]. Diabetic retinopathy (DR) is the most common microvascular complication of DM, affecting 30–40% of all diabetic patients [2]. In 2020, there were 103 million cases of DR in the world, and the prevalence is estimated to rise to 130 million by 2030 and to 161 million by 2045 [3]. DR is a leading cause of blindness in the working-age population and the elderly [3]. Diabetic macular oedema (DMO) is the most important cause of decreased vision in diabetic patients, especially in type 2 DM [4]. Vision loss in patients with type 1 DM is mainly due to complications related to proliferative diabetic retinopathy (PDR) [2]. Multimodal assessment, including fundus photography, fluorescein angiography, optical coherence tomography (OCT), OCT angiography (OCTA), and fundus autofluorescence (FAF), is the current approach in the diagnosis and follow-up of patients with DR. FAF is an imaging modality that has been used in ophthalmology for over 40 years but has recently re-emerged as clinically valuable due to technological advances [5]. FAF has been increasingly explored in recent years as an aid not only in the diagnosis and follow-up of patients with DR but also in gaining new insights into the pathophysiology and potential prognostic factors of DR and, especially, of DMO [6].

## 2. Materials and Methods

**Figure 1:**
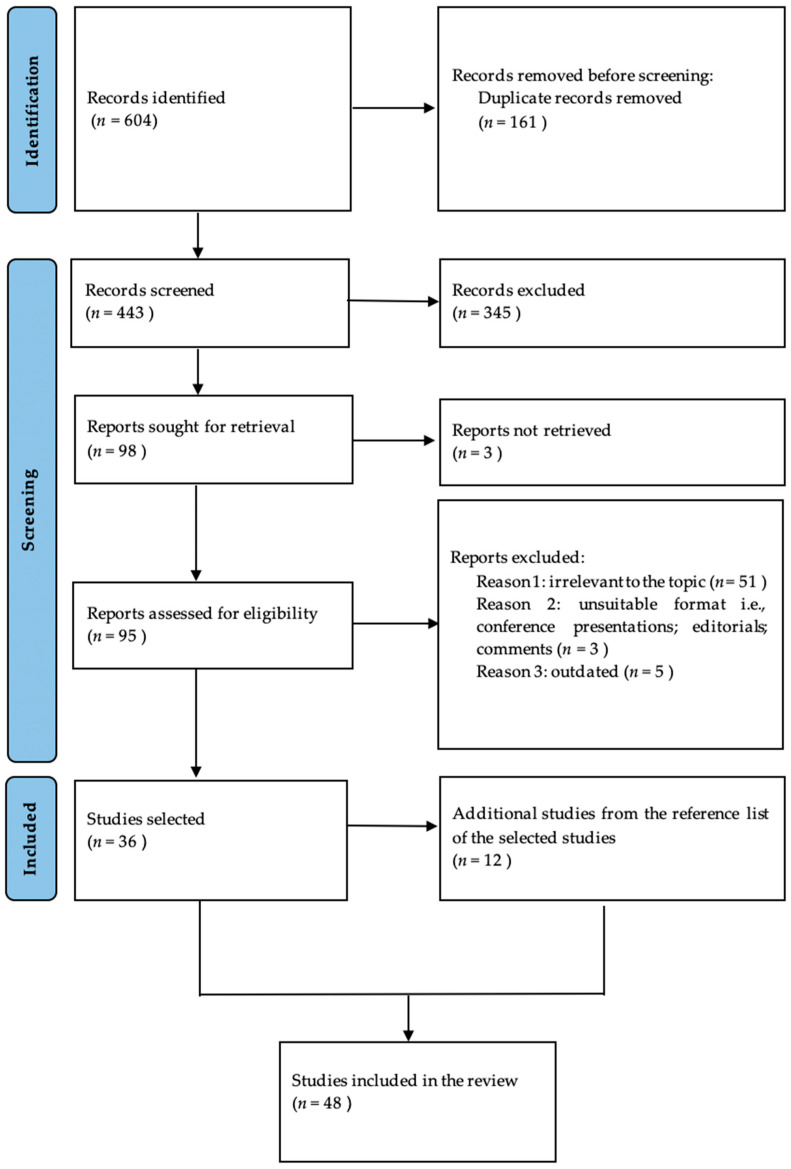
Prisma flowchart showing the article selection process.

We conducted a comprehensive literature search in the Medline electronic database using the PubMed interface. The searching process consisted of the following word combinations: in turn, all of the following, “fundus autofluorescence”, “multimodal imaging”, “multimodal assessment” AND, in turn, “diabetic retinopathy” and “diabetic macular edema”. The inclusion criteria comprised articles regarding human pathology, written in English, that were published prior to June 2024. We evaluated the title and abstract and retained the studies that investigated the use of fundus autofluorescence in retinal pathology associated with diabetes mellitus, as well as studies regarding epidemiologic, pathophysiologic, diagnostic, and therapeutic aspects related to diabetes mellitus, diabetic retinopathy, diabetic macular oedema, and fundus autofluorescence. Additional references were selected from the bibliography of the already-retained studies. Duplicates, studies irrelevant to the topic, studies on animal models, as well as conference presentations, editorials, letters to editors, and comments were excluded. After applying the inclusion and exclusion criteria, we retained 48 articles dating from 2001 to June 2024. The selection process is shown in Figure 1.

## 3. Results

### 3.1. Fundus Autofluorescence Overview

#### 3.1.1. Fluorescence and Autofluorescence

Fluorescence is a property displayed by materials possessing energy levels that are close together. In the case of these materials, after the electrons absorb a photon, they do not fall back to their original energetic level in one step, but in two steps. First, they drop to an intermediate, slightly lower level, without emitting visible energy, and then, from this intermediate level, they drop back to their original level, and in the process, emit a photon of lower energy than the one they had previously absorbed. This emitted photon has, thus, a lower frequency and a longer wavelength. This translates into the fact that fluorescent materials absorb light of a given wavelength and emit light of a longer wavelength, depending on their excitation and emission spectrum, respectively, which are characteristic for each material [7].

Autofluorescence (AF) is the inherent fluorescence of biological structures and the molecules capable of this, such as NADPH, flavins, and collagen, are termed fluorophores. Endogenous fluorophores convey fluorescent properties to the tissues [8]. In the human eye, AF is displayed by the cornea, the sclera, the crystalline lens, the retinal pigment epithelium (RPE), and the uveal tissue [9]. FAF, which is mainly derived from fluorophores at the level of the retinal pigment epithelium (RPE), is the most important application of AF in ophthalmic pathology. Changes in the AF signal are the result of either the alteration (quality or quantity) of the fluorophores or the existence of anteriorly situated compounds that either block AF or produce it themselves [10].

FAF is used in the multimodal assessment of many retinal pathologies, the most important being age-related macular degeneration (AMD) [11], macular dystrophies [12,13], retinitis pigmentosa [14,15,16], central serous chorioretinopathy [17], and macular telangiectasia [18]. In these conditions, FAF correlates with other investigations, such as the OCT, visual field assessment, and microperimetry and appears to be not only an indicator of the anatomical extension of the disease but also a severity and prognostic marker [19,20].

#### 3.1.2. Retinal Fluorophores

FAF is derived from a multitude of fluorophores [5,11,21]. These include lipofuscin, melanin, other oxidized lipids, proteins and lipoproteins, cofactors implicated in the cellular energy metabolism, and, of particular importance in the case of diabetic patients, the advanced glycation end products (AGEs) [11,22]. Proteins, such as hemoglobin and mitochondrial enzymes, display an excitation maximum around 400 nm, while flavin and flavoproteins absorb light with a maximum at 450 nm [9].

##### Lipofuscin

Lipofuscin is the major fluorophore in the human retina. Lipofuscin is a mixture of bisretinoids that result from the incomplete breakdown of photoreceptor outer segments by the post-mitotic RPE [5,6,23]. The fluorophores found in RPE lipofuscin are N-retinylidine-N-retinylethanolamin (A2E), iso-A2E, and other minor cis-isomers of A2E, all of which derive from the metabolism of all-trans-retinal in the photoreceptor cells [6]. Due to its heterogenous composition, lipofuscin has a wide range of excitation and emission spectra that range from 300 to 600 nm, and from 480 to 800 nm, respectively, with a peak of excitation at 470 nm and a maximum of emission at 600–640 nm [6,10,24].

A2E and its isomers cannot be digested by the lysosomal enzymes, leading to the accumulation of lipofuscin in the RPE lysosomes. Thus, in time, the increase in the lipofuscin content of the RPE cells is a natural consequence of aging. However, lipofuscin accumulation may also reflect an altered lysosomal activity [22]. If it exceeds a certain level, lipofuscin can cause cell disfunction and even trigger apoptosis [5]. Moreover, as many compounds that make up lipofuscin are the result of peroxidation of proteins and lipids, lipofuscin can also be regarded, at least in part, as an expression of retinal oxidative damage. The inhibition of lysosomal function, as well as the accumulation of oxidation products, leads to impaired RPE function and death, which, in turn, results in photoreceptor loss [6].

In contrast to AMD, in diabetes, lipofuscin appears to be more the result of oxidative stress and accumulates more in the microglia than in the RPE [25]. Activated microglia are part of the inflammation that is an integral part of the diabetic pathogenic process [26].

Lipofuscin distribution is the main determinant of the FAF image. Characteristically, the optic disc and the blood vessels appear dark because of the absence of lipofuscin and the blockage of the signal by blood, respectively. In the normal individual, the autofluorescence (AF) in the foveal region has a lower intensity because it is absorbed by the macular pigments (MPs) and gradually increases towards the perifoveal region [5,11].

##### Melanin

Melanin is found in the apical region of RPE cells and at the level of the choroid [27]. Unlike lipofuscin, melanin has a protective role against photooxidative stress [27]. With aging, isolated melanin is replaced by complex compounds and is found in melanolipofuscin and melanolysosomes [6]. In the case of isolated melanin, the excitation maximum is found in the range of 350–450 nm, and the emission maximum, in the range of 440–540 nm. Fluorescence intensity increases with aging, and there is a slight shift in the spectra, as melanolipofuscin exhibits an excitation maximum at 364 nm and an emission maximum at 540 nm [6,27]. Melanin is the main source of near-infrared autofluorescence (NIR-FAF) originating in the RPE and choroid [27]. In the macular region, there is a high and centripetally increasing NIR-FAF signal, due to the high melanin concentration in this area [27].

##### Advanced Glycation End Products

AGEs are the result of non-enzymatic glycation reactions, also known as “Maillard reaction”, and accumulate with progressive age and in the setting of hyperglycemia as encountered in diabetes [22]. AGEs appear to be related to cellular dysfunction at various levels, from altered expression of growth factors and extracellular matrix proteins to vasoregulatory dysfunction and apoptosis. As a result, increased levels of AGEs in the human retina promote increased vascular endothelial growth factor (VEGF) expression, death of retinal pericytes, and dysfunction of endothelial cells, all of which lead to capillary closure and retinal ischemia, on the one hand, and to vascular incompetence and leakage, on the other hand. AGEs also play an important role in Bruch’s membrane thickening, dysfunction of RPE, and altered integrity of the photoreceptor–RPE junctions [22]. AGEs display an emission maximum at 495 nm after excitation at 446 nm, leading to a green shift in the FAF as AGEs accumulate in the retinas of diabetic patients [11,28]. Moreover, AGEs appear to increase lipofuscin production and its storage in post-mitotic RPE cells [22].

#### 3.1.3. Macular Pigments

Xanthophyll MPs, which include lutein, zeaxanthin, and meso-zeaxanthin, are located in the Henle layer (outer plexiform layer) in the fovea and in the inner plexiform layer (IPL) perifoveally [29,30]. MPs have an excitation maximum at around 400–540 nm, with a peak at 460 nm and, thus, maximally absorb blue light [31]. Macular pigments have antioxidant properties, protecting the retina against photo-oxidative stress resulting from blue-light exposure. MPs reduce glare and it has been hypothesized that their concentration is a marker of photoreceptor integrity and of macular functional loss [32].

Determining the MP distribution can be achieved through autofluorescence. Macular pigment optical density assessment uses two excitation lights: one at 488 nm, which is strongly absorbed by the macular pigments, and one at 512–514 nm that is minimally absorbed. Mean images are obtained for each wavelength and then subtracted to give the MPOD at each location in the image [30,32].

Macular pigment optical density was shown to significantly correlate with HbA1c levels [30]. DM reduces MP levels even in patients without clinically detectable DR [30]. This is the result of normal cellular metabolism disruption by hyperglycemia and of increased oxidative damage [30,32]. MP levels reduce even further in patients with mild non-proliferative DR [30]. As MPs protect against oxidative stress, their depletion in the setting of diabetic inflammation potentially leads to a vicious cycle that may contribute to increased oxidative damage and to the pathogenesis of DMO [32].

#### 3.1.4. FAF Imaging Techniques

Currently, there are two main technologies that allow FAF acquisition, the conventional fundus camera and the confocal scanning laser ophthalmoscope (cSLO). As the excitation and emission spectra of retinal fluorophores usually overlay in part, both technologies select certain wavelengths for excitation and emission that elude the overlay and allow for a clear differentiation of the two [33].

The conventional fundus camera uses barrier filters for excitation and emission in order to select the desired wavelength. With this technology, the entire fundus is excited at once through an entrance aperture and AF is simultaneously detected through a separate aperture. This makes the acquired AF prone to “contamination” by scattered light originating from other planes and especially from the crystalline lens, a phenomenon termed “pseudoautofluorescence” [34]. Aperture separation in the pupil plane avoids signals from anterior ocular structures but has as drawback the reduction in the size of the apertures and, thus, in the amount of fluorescence that can be detected. Another way in which to avoid interferences is the use of longer wavelengths that do not excite the lens. Most commercially available devices use excitation wavelengths in the range of 510–580 nm and detection wavelengths in the range of 615–735 nm [33].

In contrast, the cSLO uses a monochromatic point source (a laser) and a point detector that have the same focal plane, that is, they are confocal. In this manner, scattered light is reduced to a minimum, which improves contrast by increasing the signal-to-background ratio [10]. The small excitation aperture allows for an increased area of fluorescence detection. Moreover, as the lens is only excited by a punctiform source, its autofluorescence has little interference with the FAF signal. Being punctiform, the laser has to scan the retina, moving vertically and horizontally, in order to obtain a two-dimensional image. Modern technologies allow for simultaneous FAF and OCT scans, which helps reduce motion artefacts due to eye movements [33]. The excitation wavelength is selected by choosing the appropriate laser. Depending on the type of laser used, different fluorophores are targeted, according to their excitation spectra. Blue lasers (usually at 488 nm) maximally elicit lipofuscin fluorescence but are also absorbed by the MPs. Green lasers still excite lipofuscin, while avoiding absorption by the MPs. Near-infrared lasers (usually a diode laser at 787 nm) target melanin. Barrier filters are chosen accordingly to select the desired emission wavelength [33]. Currently, there are available devices, such as the NIDEK Mirante (Nidek Co., Ltd., Gamagori, Japan), that allow the acquisition of more than one type of FAF during the same examination [35].

Apart from the optics, post-acquisition image processing also differentiates the two technologies. In conventional fundus cameras, contrast and brightness can be manually adjusted, but, as there is no standardization, this makes comparing different pictures unreliable. In the case of cSLO, multiple images are acquired and then averaged by an automated algorithm, in order to enhance contrast and image quality [10].

Both the fundus camera and the cSLO measure steady-state AF, which has a major drawback in the inability to distinguish between the different fluorophores. Time-resolved AF measures the decay in fluorescence intensity (lifetime) following pulse excitation. This allows for differentiation between fluorophores that have overlapping emission spectra and for capturing AF from otherwise weak fluorophores, which could help in detecting metabolic alterations even early on in the course of the disease [36].

This review will focus on AF obtained using steady-state techniques.

### 3.2. FAF in Diabetic Macular Oedema

The most important application of FAF in diabetic patients is the assessment of DMO. DMO represents the accumulation of fluid in the retina as a result of the disruption of the blood–retina barrier, which, in DM, has both a vascular and an inflammatory pathogenesis. The most common clinical form is cystoid macular oedema (CMO), where fluid assumes a cystic distribution that may be accessible to examination at the slit lamp. Fluid accumulates both extracellularly, in the interstitial space, at the level of the inner nuclear layer (INL) and, especially, the outer plexiform layer (OPL)—which, in the foveal region is known as the Henle layer—and, intracellularly, by swelling of the Muller cells [37].

The current gold standard for the evaluation of DMO is the OCT, and especially spectral domain OCT (SD-OCT) and swept source OCT (SS-OCT).

#### 3.2.1. Short-Wavelength FAF (SW-FAF)

On SW-FAF (488 nm), foveal DMO appears hyperAF. In particular, cystoid DMO significantly correlates with increased FAF in the fovea [29]. Multiple classifications based on the appearance of the hyperAF have been proposed (Table 1).

There are two main theories that explain the increased SW-FAF found in cystoid DMO. The first widely accepted theory has a mechanical basis and assumes that the hyperAF is the result of the displacement of the foveal MP by the intraretinal cysts, which results in an unmasking of the normal RPE fluorescence [21,29,32,37,38,40,41]. Normally, MPs absorb light within the excitation range used in SW-FAF. Thus, the observed increase in FAF is rather a “pseudoautofluorescence”, as it is not the consequence of a surplus of fluorescence, but rather the better visualization of the already-existing background FAF. This hypothesis is supported in a study performed by Waldstein et al. Their analysis of the macular pigment optical density confirmed that the increased FAF area corresponds to areas with a low concentration of MP, whereas the dark rim that surrounds them corresponds to a zone of increased MP concentration [32]. Moreover, using excitation wavelengths outside the absorption spectrum of MP (green light—580 nm) did not elicit the same increased FAF [41]. What is more, cysts located extrafoveally, where MP concentration is less important, also do not exhibit hyperAF [41].

However, studies have also found that there are patients with significant DMO who do not demonstrate the expected high level of FAF [29]. Furthermore, there are also patients who, despite the regression of the DMO on OCT, maintain persistently elevated levels of FAF [29]. This suggests that the purely mechanical displacement of MPs is not the sole explanation for the changes in FAF in DMO. Instead, it may also be related to the functionality of the foveal cells. Thus, photoreceptors could still produce and store MPs in spite of the overlying oedema, which could explain why some patients still exhibit blockage of the lipofuscin FAF. Alongside the fact that increased FAF appears to be significantly associated with markers of photoreceptor damage on OCT, lower levels of FAF in the context of important DMO may suggest the presence of residual well-functioning photoreceptors under the intraretinal fluid [29].

The second theory was proposed by Vujosevic et al., who suggested that the persistence of increased FAF even after the resolution of DMO could be explained by the persistence of activated microglia containing oxidative products that generate FAF, such as the previously discussed AGEs, that are found in excess in the retina of diabetic patients [22,38]. This may explain the pattern of spot-increased FAF, which is not associated with the presence of cystoid DMO. Spot hyperAF may also be the expression of increased photoreceptor death and the subsequent accumulation of lipofuscin in the RPE cells [21].

In terms of what decreased SW-FAF is concerned, microaneurysms and capillary dilations are readily noticeable as hypoAF lesions [21,42]. In contrast, hard exudates are less apparent [42]. Yinchen et al. described an irregular decreased FAF pattern in the presence of large amounts of hard exudates. They also postulated that this pattern may result from the complete disappearance of photoreceptors and the absent production of lipofuscin [21].

#### 3.2.2. Green FAF

Using longer wavelengths avoids absorption of the exciting light by the MPs. Although seemingly an advantage, green FAF has proven to be less useful in evaluating DMO, as it does not display the increase in FAF seen with shorter wavelengths [41,43]. Since MPs do not absorb light in the green spectrum, there is no “pseudo” FAF resulting from the unmasking of the underlying RPE fluorescence when MPs are displaced. Moreover, a study by Reznicek et al. found a poor correlation between green-light FAF intensity and OCT markers of photoreceptor integrity in diabetic patients, irrespective of the presence of DMO [43]. In a study by Bessho et al., using an excitation light of 580 nm, foveal cystic spaces did show a mild increase in FAF. The authors considered the possibility of a different fluorophore found within the cysts [41]. Overall, the utility of green FAF in DMO evaluation is limited at present.

#### 3.2.3. Near-Infrared FAF (NIR-FAF)

In NIR-FAF, the signal is mainly derived from the melanin found in the RPE and the choroid [44]. On NIF-FAF, Yoshitake et al. described three main findings: hypofluorescence at the level of the macula; a mosaic pattern, which was either granular or patchy; and a cystoid sign, representing the presence of an outline of the cystoid spaces. HypoAF on the NIR-FAF was relative and appeared in the context of serous retinal detachment and increased macular thickness. This led to the hypothesis that the decreased NIR-FAF was the result of blockage of the signal by the intraretinal or subretinal fluid [27,45]. However, the hypoAF areas did not correspond to the cystoid spaces on SD-OCT, which contradicts the previous assumption. Another possible explanation offered by the authors was that diabetes reduces the melanin content of RPE cells, which translates into a reduced NIR-FAF signal. As a result of the reduced protection offered by melanin, photooxidative stress inflicts damage on the RPE, choroid, and the intercellular junctional complexes that form the outer blood–retina barrier). Therefore, a reduction in the melanin content may be associated with an impaired outer blood–retina barrier, which, in turn, leads to fluid accumulation and neuroretinal detachment [27].

The mosaic, granular pattern appeared to be related to external limiting membrane (ELM) disruption, retinal thickness, and hyperreflective foci on OCT and to reduced VA [27,46]. The hyperautofluorescent dots in the mosaic pattern were situated at the level of or around hyperreflective foci [27]. These hyperAF more likely indicate the presence of macrophages containing melanolipofuscin and clumping of the RPE and less likely the presence of degenerated photoreceptors [46]. The hypofluorescent spots indicate reduced or absent melanin and suggest dysfunctional or atrophic RPE [46]. The association with increased retinal thickness also points to the breakdown of the blood–retina barrier. In another study, the granular pattern on NIF-FAF partially corresponded to granular hyper- and hypoAF lesions on SW-FAF, which could be explained by the fact that lipofuscin accumulation reflects a defective interaction between the photoreceptors and the RPE [46]. The authors conclude that the mosaic pattern seen on NIR-FAF is an indicator of RPE damage and of the dysfunction of the blood–retina barrier, while also being a better marker of photoreceptor degeneration than the other patterns, due to its association with ELM disruption and the presence of hyperreflective foci on OCT [27]. The cystoid sign was better related to foveal thickness and the presence of foveal cystoid spaces [27].

Ghassemi et al. found that decreased NIR-FAF may be used to detect oedematous changes in the outer retina, characterizing small and medium cysts in the ONL and INL and possibly suggesting the presence at this level of NIR-absorbing chromophores [45].

#### 3.2.4. FAF in the Multimodal Assessment of DMO

SW-FAF showed a 81% sensitivity and a 69% specificity compared to fluorescein angiography in the detection of CMO [47]. A study on 151 eyes by Vujosevic and colleagues found a correlation between SW-FAF and the cystoid pattern on OCT in 89% of cases [38]. In another study, the location of cysts on FAF and OCT correlated in 95.6% of cases [40].

Both FAF at 488 nm and MPOD appeared to correlate well to the SD-OCT, with a sensitivity of over 80% at detecting DMO, with MPDO being more specific. On the other hand, the 514 nm green FAF had low sensitivity at detecting DMO, at only 55% [32]. The amount of SW-FAF was significantly associated with OCT parameters such as increased central macular thickness (CMT), reduced ONL thickness, and the extent of inner segment/outer segment (IS/OS) disruption [29]. As the integrity of the IS/OS and ELM on OCT is an indicator of photoreceptor status, increased FAF at the level of the fovea appears to be a marker of the loss of photoreceptor integrity and of visual function [29]. However, throughout the literature, there is conflicting evidence regarding the correlation between SW-FAF intensity and retinal thickness [21,29,43]. The pattern of SW-FAF was also shown to correspond to OCT changes. Thus, in the study conducted by Yinchen and colleagues, in the normal FAF and the cystoid-increased FAF groups, the IS/OS and ELM were found to be relatively conserved, while in the spot-increased and irregular decreased FAF groups, these were disrupted. This finding was also reflected in the VA and macular sensitivity, which were found to be better in the normal FAF and cystoid FAF cases and severely affected in the spot-increased and irregular decreased FAF cases [21].

Chung at al. reported that the amount of SW-FAF was inversely proportional and significantly correlated with VA and predicted the functional visual outcome after treatment with intravitreal bevacizumab [29]. In another study, a significant correlation was found between FAF and foveal function assessed by microperimetry, showing that areas of increased FAF correspond to areas of decreased retinal sensitivity [38].

Regarding NIR-FAF, the presence of the cystoid sign was significantly associated with macular thickening and reduced VA. The mosaic pattern of hypoAF corresponded to areas of cystoid DMO and significantly correlated with central subfield thickness, ELM disruption, and the presence of hyperreflective foci on OCT and with reduced VA [27].

#### 3.2.5. FAF in Monitoring DMO Response to Treatment

SW-FAF has proven useful in evaluating the functional response to treatment in DMO. Thus, a low FAF intensity, alongside initial VA and the integrity of the ELM evaluated by OCT, was found to be a prognostic factor for the successful visual recovery after DMO remission. In this setting, a decreased FAF level may indicate, on the one hand, functional photoreceptors, capable of producing and storing MPs that block the RPE AF, and, on the other hand, less active microglia, so less inflammation and oxidative stress that damage the retinal cells [29,38]. Thus, as an indicator of cell function preservation or recovery, a reduced FAF level in DMO could serve as a prognostic factor for visual recovery after the successful treatment of DMO and may be of particular use in cases where photoreceptor integrity cannot be well assessed using OCT [29]. Although FAF alone gives limited information, being rapid and non-invasive makes it a good alternative to more invasive procedures and especially valuable in the long-term follow-up of DMO [21]. Moreover, as it is an evolving technique, new advances may provide further insights into the pathophysiology of the disease and lead to improvements in patient outcomes.

FAF has also been used to evaluate the difference between the subthreshold micropulse diode laser and the modified Early Treatment of Diabetic retinopathy Study (ETDRS) photocoagulation [48]. After conventional photocoagulation, there is early-on (within one week of laser) decreased FAF, which then turns into spots of increased FAF, most of which remain stable over months, or, less frequently, may progress to spots of decreased FAF [48]. The increased FAF may be explained by the accumulation of lipofuscin from the photoreceptors and RPE cells disrupted by the laser treatment [49]. In contrast to the conventional laser group, in the micropulse group, the FAF pattern remained unchanged even one year after the treatment. This may suggest that the subthreshold laser does not alter the photoreceptor and RPE cells and their function [48]. The evolution of scars after panretinal photocoagulation was also evaluated using FAF [49]. The scars made using a short-pulse laser displayed a slower evolution and a significantly lower expansion rate when compared to conventional laser [50]. FAF may be useful in assessing photocoagulation scar placement, especially in cases where these are not visible ophthalmoscopically, in order to guide retreatment [49].

### 3.3. FAF in Diabetic Retinal Pigment Epitheliopathy and Choroidopathy

The choroid of diabetic patients displays inner choroid atrophy (involving the choriocapillaris and Sattler’s layer), while the large choroidal vessels from Haler’s layer are engorged. On the one hand, this may explain the overall increased choroidal thickness found in these cases, which is further accentuated by choroidal circulation hyperpermeability. On the other hand, inner choroid atrophy, characterized by choriocapillaris narrowing and obstruction, leads to areas of choroidal non-perfusion and to a reduction in the amount of oxygen reaching the outer retina. Thus, diabetic choroidopathy is followed by RPE and photoreceptor dysfunction and damage. Diabetic retinal pigment epitheliopathy is characterized by RPE changes and loss of the tight junctions between RPE cells that make up the external blood–retina barrier, with subsequent subretinal fluid build-up [51].

As the RPE is the main site of lipofuscin accumulation, RPE dysfunction is reflected in SW-FAF changes. HypoAF may reflect low levels of lipofuscin, as a result of photoreceptor IS/OS and/or RPE atrophy. The hypoAF is frequently preceded by a phase of hyperAF, in which lipofuscin accumulates in abnormal RPE cells [52]. Using SW-FAF, Kang et al. described a pattern they considered indicative of diabetic retinal pigment epitheliopathy. This consisted in a diffuse granular alternation of hypo and hyperAF spots that corresponded to areas of atrophy in the IS/OS and the RPE and thinner ONL + OPL on the OCT image. Functionally, it was associated with a lower VA [51].

NIR-FAF, through the granular pattern of alternating hypo- and hyper-fluorescent dots, also points towards diabetes-related RPE dysfunction. On the one hand, hypoAF spots are suggestive of degenerated or atrophic RPE cells, and, on the other hand, hyperAF dots, situated at the level of or around hyperreflective foci as seen on OCT, likely indicate the presence of macrophages containing melanolipofuscin and clumping of the RPE. Moreover, the association of this pattern with increased central retinal thickness implies the concomitant breakdown of the blood–retina barrier. The presence of the granular changes was also correlated with reduced VA [27,46].

## 4. Conclusions

Already established as an important imaging method in retinal pathologies such as AMD, macular dystrophies, retinitis pigmentosa, and macular telangiectasia, FAF is becoming increasingly used in the evaluation of DR and, in particular, of DMO. The cSLO and the fundus camera are the two main techniques for acquiring FAF. SW-FAF mainly reflects the distribution of lipofuscin, while NIR-FAF that of melanin, although other fluorophores also contribute to the overall FAF. SW-FAF is useful for detecting cystoid DMO and correlates well with other imaging modalities such as fluorescein angiography and the OCT. In DMO, there is increased SW-FAF which has been explained by the mechanical displacement by the intraretinal fluid of the MPs that otherwise block the background AF and by the presence, in the activated microglia of diabetic patients, of oxidative products, which are by themselves fluorescent. SW-FAF correlates well with markers of photoreceptor integrity on OCT (mainly the integrity of the IS/OS and of the ELM) and with foveal function, assessed through microperimetry and VA. HyperAF both during and after the resolution of DMO is associated with poor visual function, and, as a result, SW-FAF can be used as a prognostic marker for the visual outcome after DMO treatment. Spot-increased and irregular decreased SW-FAF patterns appear to hold a particularly poor prognosis. Granular hypoAF on NIR-FAF was also associated with photoreceptor damage and decreased VA. Mosaic patterns of hyper- and hypo-AF spots on both SW-FAF and NIR-FAF indicated the presence of RPE changes that were termed diabetic retinal pigment epitheliopathy. Improvements towards a more quantitative assessment of FAF and a unification of the grading systems are future steps that could improve the utility of FAF. Moreover, further insight into the correlation between FAF and structural and functional changes in the retina, RPE, and choroid of diabetic patients could lead to a better understanding of the pathophysiology of the disease, a more timely diagnosis, and a more comprehensive evaluation of these patients. Overall, although at present, FAF alone cannot replace the other imaging techniques, it is a rapid, non-invasive tool, that can be used in the multimodal evaluation and monitoring of diabetic patients with DR and DMO and can serve as a prognostic factor for the visual function recovery.

## Figures and Tables

**Table 1:** Short-wavelength and green FAF classifications and gradings in DMO.

Study	FAF Technique and Excitation Wavelength	Classification	Study Findings (Regarding FAF)
Pece et al. [37]	cSLO 488 nm	Type 1: multilobulated Type 2: single-lobulated Type 3: mixed	Increased FAF lobules corresponded to hyporeflective intraretinal spaces as seen on OCT
Vujosevic et al. [38]	cSLO 488 nm	Normal or decreased FAF Single-spot-increased FAF Multiple-spot-increased FAF	Increased FAF (single and multiple spot) was significantly associated both with cystic changes on TD-OCT (in 89% of cases) and with a reduced retinal sensitivity, irrespective of VA
Chung et al. [29]	cSLO 488 nm	For an area of 500 µm in diameter centered on the fovea: Grade 1: no/barely visible FAF Grade 2: increased FAF of less than one-half of the total area Grade 3: increased FAF between one-half and three-quarters of the total area Grade 4: increased FAF of more than three-quarters of the total area	Lower FAF increases are associated with preserved retinal function and better visual outcomes after DMO treatment with intravitreal Bevacizumab Increased FAF correlates with OCT parameters: CMT, reduced ONL thickness and the extent of IS/OS disruption
Yinchen et al. [21]	cSLO 488 nm	Normal FAF Cystoid-increased FAF Spot-increased FAF Irregular decreased FAF	In the normal and cystoid-increased FAF groups, the IS/OS and ELM were relatively conserved, while in the spot-increased and irregular decreased FAF groups, they were disrupted. VA and macular sensitivity were better in the first two groups, while in the last two, they were severely decreased.
Hernandez-Da Mota et al. [39]	Flash fundus camera 510–580 nm	Grade 1: decreased FAF Grade 2: normal FAF Grade 3: single-spot-increased FAF Grade 4: multiple-spot-increased FAF Grade 4: plaque-like or confluent multiple-spot-increased FAF	A significant correlation exists between FAF level and macular cube average thickness on OCT before and 1 month after aflibercept intravitreal injection for DMO. No significant correlation between FAF patterns and BCVA and contrast sensitivity was found.

BCVA = best corrected visual acuity; CMT = central macular thickness; cSLO = confocal scanning laser ophthalmoscope; DMO = diabetic macular oedema; ELM = external limiting membrane; FAF = fundus autofluorescence; IS/OS = inner segment/outer segment; OCT = optical coherence tomography; ONL = outer nuclear layer; SW-FAF = short-wavelength autofluorescence; TD-OCT = time domain optical coherence tomography; VA = visual acuity.

## Data Availability

Not applicable.
